# Animal models of Huntington's disease. Pros and cons

**DOI:** 10.1016/j.bbih.2025.101149

**Published:** 2025-12-01

**Authors:** Polina Stepanova, Merja H. Voutilainen, Ove Eriksson, Dan Lindholm

**Affiliations:** a Faculty of Pharmacy, University of Helsinki, Helsinki, Finland; b Medicum, Department of Biochemistry and Developmental Biology, Medical Faculty, POB 63, FIN-00014, University of Helsinki, Finland; c Minerva Foundation Institute for Medical Research, Biomedicum-2, Tukholmankatu 8, FIN-00290, Helsinki, Finland; d Department of Biomedical and Clinical Sciences, Linköping University, Linköping, Sweden

**Keywords:** Huntington's disease, Rodent, Non-human primate, Genetic models, Neurotoxin, Neurodegeneration, Neuroinflammation, Sex differences

## Abstract

Modelling of human neurological diseases uses a plethora of ever-more sophisticated methods and approaches. For Huntington's disease (HD), which affects specific neuronal types and circuits in the brain, this has meant the use of both neurotoxic compounds and various animal models of different complexity, ranging from rodents to non-primate ones. Genetic models are classified based on the use of specific constructs including gene including gene fragments, full-length, knock-out and knock-in models. In this review, we will discuss the available animal models for HD, highlighting their pros and cons in studying the neuropathology, behavioural alterations, and biological mechanisms that prevail in HD and during the disease progression. We also highlight present knowledge gaps and difficulties to fully recapitulate the human disease. At the end we will further elaborate on current outstanding questions in HD research that warrant further studies using both animal models and patient data. This may help to guide future research and increase the translational relevance of the models to solve key questions and pave the way for better treatment options and design of drugs to alleviate the course of HD.

## Graphical abstract

The animal models employed have been described more in the text. The models are shown in yellow or blue rectangles. The particular aspects of HD preferentially addressed by each model are shown in the adjacent green ovals. At the low right, crucial methods are depicted helping to study the various research questions. The aim of the research is to increase our knowledge of HD, and to arrive at novel therapeutics for HD, for the benefit of afflicted patients and their families.

**Figure:**
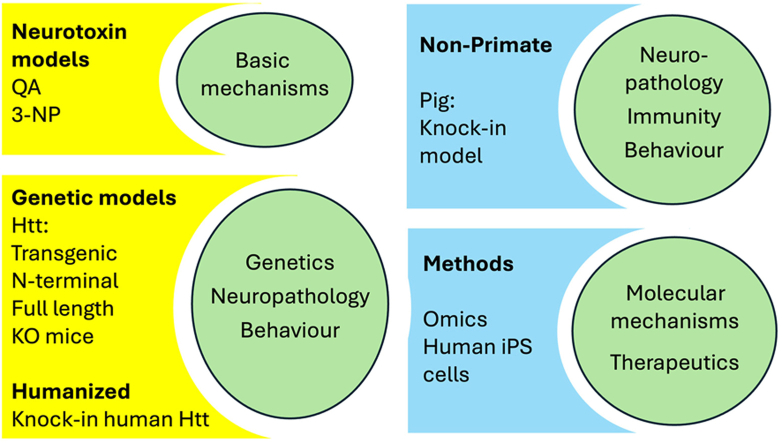

## 1 Introduction

Huntington's disease (HD) is a rare inherited disease induced by the expansion of the CAG trinucleotide repeat sequence in the *huntingtin (HTT)* gene, leading to misfolding of the huntingtin protein and the formation of huntingtin aggregates in cells. HD is characterized primarily by the triad of motor, cognitive, and neuropsychiatric symptoms in afflicted patients. Animal models can help investigate the progression, mechanisms, and molecular pathways underlying the disease. A broad range of HD models exists, ranging from neurotoxin models to those using mice with overexpression of mutant HTT under specific promoters. There are now more than 20 mouse models for HD, and around 10 rat ones. Larger animals, such as non-human primates, have also been used, especially for studying the mechanisms of HD. Although none of these models can fully outline/mimic HD, they are worthwhile in bringing novel insights into the disease pathogenesis and revealing possible treatment strategies for HD. The choice of the most suitable model depends on the actual questions being addressed. In the following review, we will discuss various models available to study HD; their pros and cons, and usefulness in research. The lesson learned is that no model is optimal and that each model has both its merits and weaknesses in mimicking HD. However, the animal models can help to answer crucial questions about the pathophysiology of HD and provide clues about possible therapies to alleviate the disease, benefiting afflicted patients and their families.

## 2 Toxin-induced rodent models for HD

In a chemical approach to HD, various neurotoxins are administered to animals *in vivo* to create lesions in specific groups of neurons, thereby mimicking certain characteristics of HD. The primary advantages of these models include their rapid onset, observable behavioral changes, and neuropathological effects, such as neuronal damage in the striatum, which is also seen in HD patients. However, these models also have notable limitations: they do not accurately replicate the chronic nature of HD, there is no expansion of CAG repeats in the *HTT* gene, and mutant huntingtin (mHTT) does not aggregate in the brain. The following neurotoxic substances have been utilized: kainic acid (KA), 3-nitropropionic acid (3-NP), malonate, and quinolinic acid (QA), which will be discussed in more detail separately.

### 2.1 Kainic acid model

Kainic acid (KA) is an agonist for a subtype of ionotropic glutamate receptors and is frequently used to model excitotoxic brain injury, particularly in the hippocampus (Sokka et al., 2007). KA leads to excessive stimulation of glutamate receptors, resulting in increased calcium and stress signaling in neurons. When KA is injected into the striatum of rats, it induces degeneration of GABAergic projection neurons in this brain region, producing changes similar to those seen in Huntington's chorea (Coyle and Schwarcz, 1976). However, KA does not selectively affect striatal neurons, which makes its use in injections a less accurate model for HD.

### 2.2 3-Nitropropionic acid model

3-nitropropionic acid (3-NP), is a toxin that inhibits mitochondrial complex II and disrupts the Krebs cycle, leading to impaired oxidative phosphorylation in neurons. It can readily cross the blood-brain barrier (BBB) (Nishino et al., 1995). 3-NP specifically targets GABAergic medium-spiny neurons, producing striatal neurotoxicity that results in motor and cognitive deficits, as demonstrated in both rats and non-human primates (Brouillet et al., 1995; Palfi et al., 1996). After two administrations of 3-NP, hyperactivity was observed, marked by increased nocturnal spontaneous locomotor activity. However, after four or more injections, hypoactivity and nocturnal akinesia were noted. Injecting 3-NP into the striatum resulted in a dose-dependent reduction of GABA, neuropeptide Y, substance P, somatostatin, as well as dopamine and its metabolites in rats; systemic administration of 3-NP did not produce these effects (Beal et al., 1993a).

In non-human primates, chronic intramuscular administration of 3-NP led to bilateral loss of neurons in the striatum, while sparing the nucleus accumbens (Palfi et al., 1996). This was accompanied by dystonia and mild dyskinesia of the limbs (Brouillet et al., 1995). The effects of 3-NP in non-human primates result in neuropathological and neurochemical changes similar to those observed in patients with HD. However, 3-NP has drawbacks, including the rapid and extensive degeneration of neurons compared to the slower disease progression seen in HD (Brouillet et al., 1995; Palfi et al., 1996). Despite these limitations, 3-NP has provided valuable insights into the roles of excitotoxicity and mitochondrial dysfunction associated with HD.

Additionally, malonate, which inhibits the tricarboxylic acid cycle, has been used to model HD in mouse (Beal et al., 1993b). This model is less characterized than the 3-NP model and will not be discussed further here.

### 2.3 Quinolinic acid model

N-methyl-D-aspartate (NMDA) receptors are widely distributed throughout the brain, including regions such as the hippocampus, pallidal formation, and striatum. Quinolinic acid (QA), an agonist of the NMDA receptor, has been utilized in excitotoxic models for HD (Schwarcz and Kohler, 1983). However, QA cannot cross the BBB and must be injected directly into the brain parenchyma (Foster et al., 1984). Tryptophan, a precursor of QA, crosses the BBB and increase QA levels in the brain (Chen and Guillemin, 2009; Hestad et al., 2022).

In rats, endogenous QA is found in various brain regions, including the striatum and frontal cerebral cortex. Its concentration in the brain is age-dependent, with levels increasing as the rat ages (Moroni et al., 1984). In human postmortem tissue, QA has been detected in the caudate nucleus and the frontal cortex (Wolfensberger et al., 1983). High levels of QA were particularly observed in early-grade (grade 0/1) HD patients, but not those with late grades (3 or 4) (Guidetti et al., 2004).

Microglia and infiltrating macrophages are the primary producers of QA in the brain. Although human fetal brain microglia can synthesize QA, they do so at lower levels than macrophages. This suggests that the activation of microglia may contribute to QA production (Heyes et al., 1997; Guillemin et al., 2003). Astrocytes do not produce QA but are involved in its metabolism (Guillemin et al., 2001, 2005) through the enzyme, quinolinic acid phosphoribosyltransferase. Low levels of this enzyme are also present in some neurons (Kohler et al., 1987), as well as in microglia and macrophages (Guillemin et al., 2001). Under normal conditions, basal concentrations of QA in the human and rat brain, as well as in cerebrospinal fluid (CSF), are less than 100 nM (Chen and Guillemin, 2009; Pierozan et al., 2010; Lugo-Huitron et al., 2013). However, under pathological conditions, QA levels can increase significantly, ranging from 500 to 1200 nM (Guillemin, 2012).

Experimental studies have shown that the dose of QA required to induce neurotoxicity in the rat striatum ranges from 150 to 225 nmol. A dose of 75 nmol of QA does not significantly affect locomotor activity, while a dose of 300 nmol is lethal (Sanberg et al., 1989).

Bilateral striatal injections of QA (Perez-De La Cruz et al., 2009) produce behavioral changes in rats, such as increased rearing and grooming in response to stressful situations (Scattoni et al., 2004). Grooming behavior is modulated by D1-like receptors, while rearing behavior dependents on both D1-and D2-like receptors (Xu et al., 1994; Scattoni et al., 2004). Rats with bilateral QA-induced medial striatal lesions exhibit impaired memory retention, decreased cognitive flexibility and spatial learning difficulties, with visuospatial impairments evident in tests, such as the Morris water maze one (Furtado and Mazurek, 1996). These cognitive dysfunctions resemble those observed in HD patients (Heindel et al., 1989).

Neuropathologically, striatal injections of QA selectively damage GABAergic and substance P neurons, while preserving neuropeptide Y and somatostatin-containing medium spiny neurons. These alterations mirror those seen in HD, making the QA toxin model a useful tool simulating cell degeneration associated with the disease (Beal et al., 1986; Lugo-Huitron et al., 2013). It has been reported that QA reduces GABA and substance P concentrations by approximately 40 % in the rat, while increasing somatostatin (by 115 %) and neuropeptide Y (by 41 %) at six-months post-lesion, with no changes observed in the cerebral cortex. Similar changes have been reported in the rat brain at six months post-lesion following QA treatments (Beal et al., 1991).

QA can have multiple effects on neurons, influencing metabolism (Shear et al., 1998b) and the cytoskeleton by affecting tau phosphorylation (Pierozan et al., 2010), which may contribute to neuronal damage. As discussed, the use of QA can accurately mimic several neurochemical features of HD. However, this model has limitations, including the rapid progression of neuropathology and the absence of mHTT inclusions commonly found in HD brains.

### 2.4 Comparison of the 3-NP and QA rodent models

Comparing the most widely used neurotoxins, QA and 3-NP, in modeling HD, notable differences emerge. Intrastriatal administration of QA in rats creates a substantial a rea of metabolic deficiency, while intrastriatal injection of 3-NP leads to the formation of necrotic cavities within the striatum. Both QA and 3-NP result in a reduction of striatal area and a decrease in Nissl- and NADPH-diaphorase-immunoreactive neurons in the central area of the lesion.

When injected intrastriatally into rats, both QA and 3-NP affect motor and learning abilities (Shear et al., 1998a). However, 3-NP has a more pronounced impact on motor function, reducing motor activity by approximately 80 % on the first day post-lesion and by 65 % one-week later (Perez-De La Cruz et al., 2009). Behaviorally, 3-NP disrupts working and reference memory to a greater extent than QA (Shear et al., 1998a), while QA appears to selectively impair reference memory (Emerich et al., 1997).

The QA-induced lesions in rats result in hyperactivity and an inability to use forelimbs, as measured by the number of pellets eaten in the staircase test (Sanberg et al., 1989; Emerich et al., 1997). In contrast, the administration of 3-NP leads to hypoactivity (Koutouzis et al., 1994). In non-human primates, a comparison can be made between the intramuscular administration of 3-NP and bilateral intrastriatal injections of QA (Roitberg et al., 2002). QA resulted in neuronal loss and bilateral damage in the striatum while sparing glial cells, whereas 3-NP affected a smaller area in the dorsolateral putamen. The bilateral injections of QA induced hyperactive behavior in non-human primates, particularly at night, whereas 3-NP-lesioned animals exhibited spontaneous dyskinesia and dystonia.

## 3 Genetic mouse models for HD

Genetic models are valuable tools for studying the pathophysiology and molecular characteristics of HD, serving as a foundation for the development of novel therapeutics. Generally, the mouse genetic models can be categorized into three groups based on the genetic constructs used.

1) Models that carry a short toxic mutant N-terminal fragment of HTT with CAG expansions – such as R6/1, R6/2, and N171-82Q (Mangiarini et al., 1996; Davies et al., 1997; Schilling et al., 1999).
2) Models that express full-length human mHTT including YAC128, BACHD and BAC-225Q (Slow et al., 2003; Van Raamsdonk et al., 2005b; Gray et al., 2008).
3) Humanized HD mouse models, like Hu97/18 and Hu128/21 knock-in models (Southwell et al., 2017), which contain expanded CAG repeats, along with other knock-in models such as the HdhQ50, HdhQ72, HdhQ80, HdhQ92, HdhQ140, Hdh150Q, and zQ175 (Menalled et al., 2003, 2012). The onset, progression and severity of impairments vary depending on the genetic construct used. A significant decline in muscle strength is observed in category 1) N-terminal HD mice, but this is not seen in category 2) or category 3) mice. These models also exhibit several behavioral and neuropathological characteristics commonly associated with HD, and some show mHTT inclusions in the brain, albeit to varying degrees.

### 3.1 N-terminal mutant huntingtin fragment mouse models for HD

The N-terminal mHTT mouse transgenic models, including R6/1, R6/2, and N171-82Q express a toxic N-terminal human mutant HTT fragment, which is particularly harmful to cells. These N-terminal mHTT models incorporate two copies of the wild-type *HTT* gene and one mutated copy, resulting in a rapid onset of motor symptoms that progress more quickly than those in the full-length HD mouse models (Stack et al., 2005; Crook and Housman, 2011).

#### 3.1.1 R6/2

The R6/2 model is the most widely used transgenic rodent model for HD. It expresses exon-1 of the human huntingtin gene (HTT) with 144–150 polyglutamine (CAG) repeats under the control of the human HTT promoter (Mangiarini et al., 1996; Davies et al., 1997). Significant symptoms in R6/2 mice appear as early as 9–11 weeks of age, including impaired motor performance and dystonic movements, which typically lead to death between 10 and 14 weeks (Mangiarini et al., 1996; Stack et al., 2005; Wang et al., 2008). The severe phenotype and rapid onset of symptoms make the R6/2 mice particularly valuable for drug screening.

At later stages of the disease, approximately 25 % neuronal loss occurs in the striatum of R6/2 mice (Mangiarini et al., 1996; Stack et al., 2005). At 6 weeks of age, the R6/2 mice show few changes in brain region volumes; however, by 14 weeks, the volumes of the cortex, striatum and hippocampus are significantly reduced compared to control wild-type (WT) mice (Cepeda-Prado et al., 2012). By 18 weeks, R6/2 mice also exhibit enlargement of the lateral ventricles and lateral globus pallidus, along with increased body weight in R6/2 males compared to WT males (Sawiak et al., 2009).

Neurodegeneration in R6/2 mice correlates with an increase in the number and size of mHTT aggregates. These aggregates are present from postnatal day 1, gradually increasing in both number and size (Stack et al., 2005). Neuropil aggregates are more prominent than neuronal intranuclear inclusions (NIIs), with approximately 60 % found in the axons and axonal terminals (Li et al., 1999). Axonal pathology is noted within the corpus callosum of R6/2 mice, paralleling the degeneration of interhemispheric connections observed in HD patients (Crawford et al., 2013; Gatto et al., 2019). Neuropil aggregates are more prevalent in the cortex than in the striatum of R6/2 mice, with quantities increasing in both areas as disease progresses. Few neuropil aggregates are ubiquitin-positive, whereas the majority of NIIs are (Gutekunst et al., 1999; Li et al., 1999). NIIs are less common in early-stage HD brains, whereas neuropil aggregates are widely dispersed across brain regions.

Regarding the mechanisms for cell degeneration, apoptotic neurons are not significantly detected in the brains of R6/2 mice (Turmaine et al., 2000), However, dark neurons associated with mHTT are observed at 10 weeks of age (Yu et al., 2003). Additionally, there is an increase in levels of 3-hydroxykynurenine (3-HK), a precursor of QA, in the cortex, and striatum of these mice (Guidetti et al., 2006).

Behaviorally, the R6/2 mouse line is sensitive to the development of audiogenic seizures of varying intensities (Cepeda-Prado et al., 2012). Cognitive performance deteriorates with age, with no learning impairments observed in younger mice but significant regression noted in 10 week-old animals (Lione et al., 1999). Anxiety levels increase in R6/2 mice starting from 4.5 weeks of age (Hickey et al., 2005). Additionally, R6/2 mice experience visual dysfunctions, including retinal degeneration and the accumulation of NIIs within neurons (Helmlinger et al., 2002). NIIs are also found in the pancreas of R6/2 mice as early as 6 weeks of age, increasing in number at later stages (Andreassen et al., 2002). These aggregates are particularly concentrated in β-cells, leading to hyperglycemia and development of diabetes mellitus (Bjorkqvist et al., 2005). Correspondingly, insulin levels are decreased in 12 week-old R6/2 mice, impairing both basal and glucose-stimulated insulin secretion (Bjorkqvist et al., 2005). Peripheral inclusions are also present in other organs, including the heart, liver, kidneys, and adrenal glands (Moffitt et al., 2009). Disturbances in sleep and circadian rhythm are observed in some HD patients, affecting both females and males. R6/2 mice demonstrate changes in circadian activities that worsen with disease progression and affects the normal night-day activity patterns. An altered gene expression in the suprachiasmatic nuclei in the brain controlling circadian rhythms are likely to contribute to these changes (Morton et al., 2005).

#### 3.1.2 R6/1

In the R6/1 model, exon-1 of human *HTT* containing115 CAG repeats is expressed under the human *HTT* promoter (Mangiarini et al., 1996; Davies et al., 1997). Generally, R6/1 mice exhibit slower disease progression with a later onset of symptoms (at 15–21 weeks) compared to R6/2 mice, and they have a lifespan of around 32–40 weeks (Mangiarini et al., 1996; Yu et al., 2003). Behavioral changes, such as foot clasping, become evident around week 30, increasing over time, while gait and locomotion impairments appear by week 38. R6/1 mice also display a decrease in rearing and an increase in grooming, along with a significant loss of D1-like and D2-like receptors in the striatum (Clifford et al., 2002). By 16 weeks, they exhibit loss of retinal ganglion neurons and retinopathy (Helmlinger et al., 2002).

In R6/1 mice, nuclear and neuropil aggregates are present in the striatum alongside dark neurons (Yu et al., 2003). By 30 weeks, volumes of the striatum, corpus callosum, and other regions are significantly lower in symptomatic R6/1 mice compared with control animals (Gatto et al., 2021). Unlike R6/2 model, R6/1 mice do not develop diabetes, however, they demonstrate abnormal glucose control and accumulate mHTT aggregates, leading to reduced β-cells mass in the pancreas (Josefsen et al., 2008).

There is also a notable decrease in striatal DARPP-32 immunoreactivity in the R6/1 mice beginning at 5 months of age (van Dellen et al., 2000). Intrastriatal QA injections lead to cell death in control mice, whereas presymptomatic R6/1 mice show protective effect (Hansson et al., 1999).

#### 3.1.3 N171-82Q model for HD

The N171-82Q mice express exons 1 and 2 of the human *HTT* gene with 82 polyglutamine (CAG) repeats, regulated by the mouse prion promoter (Schilling et al., 1999). This results in higher levels of the *mHTT* transcript in the brain compared to the R6/2 mouse model (Wang et al., 2008).

N171-82Q mice display a progressive neurological phenotype that is slower and less severe than that observed in R6/2 animals. The first symptoms appear between 2 and 3 months of age, marked by a loss of body weight (Schilling et al., 1999). These mice have a lifespan of 16–22 weeks, with female mice living longer than males and losing weight at a later stage. While brain weigh decreases equally in both sexes, there is a reduction in neuronal numbers in the striatum of N171-82Q (McBride et al., 2006; Stepanova et al., 2023).

Behaviorally, N171-82Q mice exhibit progressive limb clasping, tremors, motor impairments and reduced latency to fall, as well as decreased grip strength (Schilling et al., 1999; McBride et al., 2006; Stepanova et al., 2023). Gender differences have been observed, with clasping and latency to fall appearing in males around 9–10 weeks and in females from the 12th week (Stepanova et al., 2023). Furthermore, significant impairments in working and reference memory, as well as learning, were noted at 14 weeks of age in the N171-82Q mice (Ramaswamy et al., 2007).

An age-dependent increase in EM48-positive mHTT aggregates was observed in the cortex and striatum of N171-82Q mice, including the presence of nuclear aggregates (Stepanova et al., 2023). These EM48-immunoreactive aggregates were found in lower density and were localized to neuronal cells, with fewer aggregates in the white matter compared to the R6/2 and R6/1 mice (Yu et al., 2003; Wang et al., 2008). In contrast, immunoreactive Glial Fibrillary Acidic Protein (GFAP), a marker for reactive gliosis, was more pronounced in N171-82Q mice compared to the R6/2 and R6/1 models (Yu et al., 2003), with increased GFAP staining evident in the striatum, cortex and hippocampus.

Regarding the mechanism of cell demise, there was a significant increase in TUNEL-positive neurons in the cortex and striatum of N171-82Q mice aged 3–5 months (Turmaine et al., 2000; Yu et al., 2003). Additionally, mHTT inclusions were found in neuronal nuclei undergoing apoptosis (Yu et al., 2003).

Peripheral pathology, including muscle atrophy and tissue degeneration, is prevalent in N-terminal mHTT mouse models, affecting heart rate (Griffioen et al., 2012). N171-82Q mice demonstrated hypothermia, an increase in brown adipose tissue (BAT) and elevated fasting blood glucose levels starting around 80 days of age (Andreassen et al., 2001). Hyperglycemia was also observed in these mice with mHTT inclusions present in the pancreas (Martin et al., 2009).

In summary, compared to other transgenic models, N-terminal mHTT mouse models exhibit relatively early onset of symptoms. The N171-82Q model enables researchers to study disease progression on a more manageable time scale, effectively reducing both drug screening duration and costs.

### 3.2 Full-length mutant huntingtin mouse models for HD

Full-length mHTT transgenic mouse models are defined by the expression of the human *HTT* gene in mice. In the YAC128 mouse model, a yeast artificial chromosome (YAC) with 128 CAG-expanded repeats is expressed (Slow et al., 2003). Levels of QA and 3-HK levels are elevated in the cortex and striatum of these 12-month-old mice (Guidetti et al., 2006). The YAC128 mice are often compared to BACHD mice, which express a bacterial artificial chromosome (BAC) containing 97 CAG repeats. Unlike the CAG repeats in the YAC128 mice, BACHD mice exhibit a mixture of CAG and CAA repeats, which can influence the sequence's stability (Gray et al., 2008; Gu et al., 2022). Both mouse models show a gradual increase in disease pathology; brain atrophy is apparent at 9 months in the YAC128 mice and at 12 months in the BACHD mice (Slow et al., 2003; Van Raamsdonk et al., 2005b; Gray et al., 2008). mHTT aggregates are observed in both mouse models, but they appear later and in lower numbers compared to N-terminal mHTT mouse models (Slow et al., 2003; Gray et al., 2008).

In the YAC128 model, NIIs are present in the cortex, striatum, and hippocampus, whereas BACHD mice demonstrate fewer NIIs, primarily affecting the cortex and striatum (Van Raamsdonk et al., 2005a; Gray et al., 2008). The NIIs in YAC128 mice resemble those found in HD patients.

Notably, the size and number of Iba1-positive microglia are slightly increased in the striatum, while the number of primary microglial processes is significantly decreased in the 12-month-old YAC128 mice. These findings align with the morphological changes observed in microglia during disease progression (Franciosi et al., 2012) and are consistent with observations made in human brain tissue of HD patients, as reviewed by Palpagama and colleagues (Palpagama et al., 2019).

Behaviorally, the BACHD mice exhibit significant and progressive motor deficits beginning at 2 months of age, while these deficits are milder and appear later in the YAC128 mice. (Slow et al., 2003; Van Raamsdonk et al., 2005b; Gray et al., 2008). Cognitive impairments in the YAC128 mice start at 2 months and worsen over time (Van Raamsdonk et al., 2005b). This progression resembles the cognitive deficits observed in both pre-symptomatic and symptomatic HD patients, including impairments in procedural learning, attention, long-term memory and memory retrieval (Knopman and Nissen, 1991; Kobal et al., 2014; Jellinger, 2024).

Both YAC128 and BACHD mice show an increase in body weight starting at 2 months (Van Raamsdonk et al., 2006; Gray et al., 2008). In the YAC128 mice, the weight increase is primarily in peripheral organs, while brain weight is significantly lower compared to WT mice (Van Raamsdonk et al., 2006). In BACHD mice, the forebrain weighs 20 % less than that of WT mice (Gray et al., 2008). Regarding gender differences, BACHD males weigh significantly more, and they also have severe circadian dysfunctions compared with BACHD females (Kuljis et al., 2016). The changes warrant more studies in the future.

Recently, a novel full-length HTT mouse model for HD, referred to as BAC226Q, was developed. This model expresses the full-length human HTT with 226 CAG repeats under the control of the human HTT promoter (Shenoy et al., 2022). Analyses of the BAC226Q mice revealed an early onset of disease, characterized by progressive motor impairment and a gradual weight loss of 40 % by 11 months of age, leading to a shortened lifespan. These mice also exhibited cognitive deficits along with neuropathological changes in the striatum and cortex, including increased neuronal death, gliosis, atrophy, and the formation of mHTT aggregates in the brain. These promising findings necessitate further studies utilizing the BAC226Q mice in the future.

### 3.3 Humanized HD mouse models

The humanized HD mouse model, Hu97/18, is created by intercrossing YAC18 and BACHD mice with a deletion of the mouse *huntingtin* gene (*Hdh*). The Hu128/21 mice were generated through intercrossing on the Hdh knock-out background with YAC128 and BAC21 (Southwell et al., 2017). The levels of human wild-type and mutant HTT protein in the cortex are similar in both Hu128/21 and the parental YAC128 mice. Both the Hu128/21 and Hu97/18 mice develop motor deficits and depressive-like behaviors as well as learning impairments. Evidence of forebrain atrophy is present, along with a decrease in brain tissue in the striatum and corpus callosum. In Hu128/21 mice, striatal Em48-positive inclusions become visible in the striatum by 3 months of age and are associated with the loss of striatal DARPP-32 immunoreactivity. Additionally, Hu128/21 model shows a gradual increase in body weight, particularly in female mice consistent with the findings in the YAC128 mice (Southwell et al., 2015). The same trend is observed in Hu97/18 female mice, indicating a possible gender-dependent metabolic dysfunction that may also contribute to the differences noted in motor performance between sexes (Southwell et al., 2013). The humanized HD mice express human-specific genetic mutations that can rather accurately model different aspects of the human disease. So far these mice have been less frequently used in HD research, but in the long-run they can give valuable insights about mechanisms and neuropathology of the disease in the future.

### 3.4 Mutant huntingtin knock-in mouse models for HD

In the mHTT knock-in (KI) models, genetic constructs of the mouse *Hdh* gene incorporate different disease-causing CAG repeats, with expression driven by the endogenous *Hdh* promoter. (Shelbourne et al., 1999; Lin et al., 2001; Menalled et al., 2012). The number of CAG triplets in these models can vary from 50 to 175 repeats, with longer repeats being more unstable, mimicking the CAG instability observed in HD (Shelbourne et al., 1999; Lloret et al., 2006). This instability is region-specific, as the CAG repeat number increases particularly in the striatum and cortex (Menalled and Chesselet, 2002). The KI mouse models with shorter CAG repeats, such as HdhQ50 and HdhQ92 mice, exhibit fewer motor disturbances. In contrast mice with longer CAG repeats show these disturbances at an early age (Wheeler et al., 2000; Menalled and Chesselet, 2002; Menalled et al., 2012). Compared to other full-length mHTT models, the KI mice show a later onset of neuropathological changes but display a broader range of behavioral impairments (Lin et al., 2001). For instance, Hdh150Q mice exhibited cognitive impairments at 22 months of age (Woodman et al., 2007), while HdhQ72 and HdhQ80 mice displayed aggressive behavior starting at 3 months (Shelbourne et al., 1999).

Axonal changes are present in the KI models, but there is no loss of neurons or enhanced gliosis (Menalled and Chesselet, 2002; Yu et al., 2003). However, the Hdh150Q mice demonstrated an increase in GFAP immunoreactivity in the striatum at 14 months of age (Yu et al., 2003), although this was less pronounced than in the N171-82Q mice. Neuropil aggregates and NIIs are observed in specific brain regions in the KI mice (Lin et al., 2001; Menalled and Chesselet, 2002; Yu et al., 2003). In the Hdh150Q model, these aggregates appear in the striatum and hippocampus by 6-months of age and increase in frequency over time. A similar pattern is observed in 12-week-old R6/2 mice (Woodman et al., 2007), but the R6/2 line exhibit a greater density of aggregates (Yu et al., 2003). The HdH150Q mice also exhibit some aggregates in peripheral tissues, such as the liver, adrenal glands, and pancreas (Moffitt et al., 2009).

The zQ175 KI mouse line was introduced in 2012 due to spontaneous expansion of CAG repeats in the Q140 KI mouse colony (Menalled et al., 2012). The earliest animals in the cohort had 175 CAG repeats, which later increased to 188 CAG repeats (Menalled et al., 2003). Homozygous zQ175 mice displayed behavioral changes including body tremors, twitching, posture and gait abnormalities, and weight loss. These mice also showed impairments in learning and memory (Menalled et al., 2012). The zQ175 female mice do not show an early hypertrophy, and the changes in the striatum, globus pallidus, and cerebellum are observed only in the male mice (Zhang et al., 2020). Overall, the KI mouse models are highly relevant for studying HD and can mimic the human condition. However, one drawback of these models is the very late onset of neuropathology and motor abnormalities, which complicates the screening of novel drug candidates.

### 3.5 Transgenic rat models for HD

There are two commonly used transgenic rat models for HD.

1) the N-terminal mHTT fragment transgenic rat model (tgHD51). This model expresses 51 CAG repeats under the control of the endogenous rat *HTT* promoter (von Horsten et al., 2003).
2) the full-length mHTT rat model (BACHD). This model features 97 mixed CAA-CAG regulated by a human *HTT* promoter (Yu-Taeger et al., 2012).

Studies of tgHD rats have shown a loss of striatal volume and enlargement of the lateral ventricles in 8-month-old animals (von Horsten et al., 2003). By 12 months, accumulation of mHTT was observed in the nuclei and neuropil, mainly within the ventral and caudal striatum, with some aggregates found in the cortex as well (von Horsten et al., 2003). Levels of dopamine, tryptophan, and xanturenic acid were reduced in the striatum of tgHD rats, while the level of 3,4-Dihydroxyphenylacetic (DOPAC) remained constant (von Horsten et al., 2003).

In the BACHD rats, mHTT aggregates were predominantly found in the cortical neuropil, but not in the striatum, from 3 months of age onwards (Yu-Taeger et al., 2012).

The tgHD rats displayed a gradual onset of motor deficit, gait abnormalities, and balance impairments. Their body weight declined over time, and by 24 months of age, they experienced a rather rapid weight loss associated with muscular atrophy, ultimately leading to death (von Horsten et al., 2003). They exhibited chorea-like movements (von Horsten et al., 2003; Cao et al., 2006), but did not show clasping, resting tremors, vocalization, or seizures. In contrast, BACHD rats presented with early-onset motor deficits (Yu-Taeger et al., 2012), and exhibited impaired motor coordination (Novati et al., 2020).

Cognitive assessment revealed that the tgHD rats have difficulties with spatial learning starting at 10 months of age, and this worsened over time (von Horsten et al., 2003; Cao et al., 2006). The BACHD rats also demonstrated a progressive decline in cognitive function, including learning deficits beginning at 6 months of age (Clemensson et al., 2019). Anxiety-like behaviors were observed in both 2-month-old tgHD rats (von Horsten et al., 2003), and in BACHD rats, with these behaviors worsening as the rats aged. (Lamirault et al., 2020). The older BACHD rats displayed reduced motivation, which was associated with motor impairments and increased anxiety (Lamirault et al., 2020).

There is no clear evidence of gender differences between BACHD male and female rats with respect to motor and cognitive deficits, or neuropathology. In contrast, the tgHD rats display gender differences (Bode et al., 2008) with tgHD males having more severe motor impairments and a greater loss of DARPP-32-positive medium-sized spiny neuronal (MSN) compared with the females. One contributing factor for this could be hormonal, as estrogen can be neuroprotective and estrogen receptors are expressed by striatal neurons. However, this issue will require more detailed studies.

### 3.6 Knock-out HD mouse models

In the knock-out (KO) mouse model of HD, the mouse *HTT* gene is genetically deleted, resulting in the absence of functional protein. Mice lacking *HTT* exhibit abnormal brain development and experience embryonic lethality by day 8.5, indicating that wild-type *HTT* plays a role in embryonic development (Duyao et al., 1995; Zeitlin et al., 1995). Consistent with this, increased cell death has been observed in nullizygous embryos, suggesting that wild-type *HTT* may have a protective role in cells (Zeitlin et al., 1995). Studies on *Htt* conditional KO mice, in which *HTT* is inactivated in the brain and testis during the early postnatal period, have revealed increased neurodegeneration, motor deficits and impaired spermatogenesis, ultimately leading to a shorter lifespan for these animals (Dragatsis et al., 2000).

In contrast, the inducible knock-out of *HTT* in the adult mouse brain does not produce any neuropathological deficits or significant changes in body weight or behavior (Wang et al., 2016). However, the depletion of HTT throughout the entire body, using a conditional ubiquitous KO model, resulted in acute pancreatitis and subsequent cell death (Wang et al., 2016).

Interestingly, the inactivation of the *Hdh* gene in conditional heterozygous ubiquitous KO mice led to progressive motor deficits, induing gait abnormalities, tremors, decreased locomotion, a shortened lifespan and progressive neuropathology (Dietrich et al., 2017). The discrepancies observed regarding the effects of *HTT* elimination may be related to the specific background of the mouse line or the levels of endogenous *HTT* present during the genetic manipulations.

Overall, studies involving the deletion of the mouse *HTT* gene at various developmental stages and using mice with different genetic backgrounds have significantly enhanced our understanding of the role of *HTT* in cells. However, knock-out mouse models with a complete and constitutive elimination of *HTT* may not be ideal for detailed studies of the neuropathology and potential treatments modalities for HD.

## 4 Neuroinflammation and HD

Neuroinflammation is a common feature in many neurodegenerative diseases, including HD (Mason and McGavern, 2022). The presence of mutant *HTT* in both brain and immune cells disrupts cell signaling and promotes inflammatory responses by increasing the expression of immune mediators in the brain and periphery (Palpagama et al., 2019). In HD, pro-inflammatory cytokines such as IL-6, IL-8, TNF-α are elevated in the plasma during the early stages of the disease, and similar increases in these cytokines have been observed in postmortem striatal tissue from HD patients (Bjorkqvist et al., 2008). Some of these cytokines are also upregulated in HD mouse models, such as the R6/2 and HdH150Q models (Bjorkqvist et al., 2008). For instance, in the YAC128 mouse model, IL-6 levels were significantly higher at 12 weeks, indicating an early phase of HD (Bjorkqvist et al., 2008).

Furthermore, zQ175 mice exhibited significant cytokine expression in the spleen, with the elevations in IL-10, IL-12, IL-17 and TNF-α (Pido-Lopez et al., 2018). These studies collectively demonstrate that the inflammatory changes are recapitulated in HD mouse models. The roles of various cytokines and the interaction between brain microglia and peripheral cells in exacerbating neuroinflammation in HD will require further investigation. In this context, the cytokine IL-17, produced by pro-inflammatory T helper (Th17) cells, is particularly noteworthy. Studies have indicated enhanced Th-17.1 cell activity in the CSF of early HD patients (von Essen et al., 2020). Recently, increased expression of IL-17-signaling-related genes has been observed in the brains of the knock-in (150Q repeat HD KI) pig models of HD compared to controls (Jia et al., 2023). These pigs displayed brain tissue changes resembling those seen in HD patients, including reactive gliosis, axonal degeneration and loss of striatal neurons. In contrast, the HD KI (140Q repeats) mouse model exhibited milder neuropathology and no increase in IL-17-regulated genes in the brain. Notably, administration of IL-17 to the striatum of HD KI mice resulted in an enhanced gliosis and synaptic deficiencies compared to vehicle injections (Jia et al., 2023). These findings highlight some important differences between pig and mouse HD models, suggesting that larger animals may better mimic the neuropathology observed in HD patients. Future studies on IL-17 signaling and other cytokines in HD models, and the interplay between immune and glial cells may reveal important insights about their roles in protein aggregation and the loss of neurons occurring in HD.

## 5 Gender differences and HD

Clinical observations and some epidemiological studies support the view that there is a gender difference in HD with females having a higher disease prevalence, a more severe phenotype, and a faster disease progression. These differences have not been observed in all studies, and the mechanisms involved have remain largely unclear. It is thought that an enhanced immune response in females may contribute to some gender differences but other factors are also likely to play a role. For a more thorough discussion on this topic see a recent review (Risby-Jones et al., 2024). Generally it can be said that the gender issues have been dealt with rather shortly and in only few work on animal model for HD. This is also evident in our description of the different rodent models above. The studies done have further focused mainly on motor performance and signs of disease and less on non-motor symptoms. HD patients frequently experience symptoms, such as depression, apathy, and aggression, which also affect patients' well-being, and life quality. Unfortunately, rodent studies addressing these points is limited, highlighting a significant gap in HD research that needs to be addressed. There is also currently no universal animal HD model which can address all these issues requiring more sophisticated analyses. Future studies and additional models are therefore required in order to clarify the gender and non-motor issues in more depth, to increase knowledge and bring help for the patients afflicted.

## 6 Conclusions and outstanding questions in HD research

Animal models can prove very useful revealing basic molecular mechanisms of human neurological diseases such as for HD. However, each model has its inherent strength and weaknesses and the translational relevance to the human disease is not perfect. This varies also depending on which research question or topic the model is set to address (see Graphical Abstract). In Table 1 we present a list of some **outstanding questions** in HD research that will require further studies both using animal models and data from human patient to be dealt with successfully. It is clear that such endeavors cannot be tackled in a short time and without great efforts. However, the development and availability of advanced emerging techniques can support and make such efforts more possible. In this respect, we envision that the use and integration of research data from multiple sources (genomics, proteomics, metabolomics, single-cell analyses) can help to uncover issues on basic mechanism, disease vulnerability and altered signaling networks in HD. In parallel, work on human-derived iPSC-based neuron and organoid models will increase the translational relevance of the findings, and further help in design of modifying drugs and better disease management.

**Table 1:** Outstanding questions in HD research related to the use of animal models.

Disease initiation	Early pathogenic mechanisms	Disease drivers	Disease modulators
**Selective vulnerability of striatal neurons**	**Mutant Htt misfolding aggregation clearance**	**Somatic CAG repeat instability**	**To lower or correct mHTT levels** **Allele-selective suppression and editing**
**Role of non-neuronal cells** **Astrocytes, microglia oligodendrocyte** **in disease**	**Autophagy** **Proteasome** **Stress granule**	**Altered gene expression and cell signaling**	**Symptomatic and neurorestorative treatments:** - Trophic factors - Cell therapies - Small molecules - brain stimulation
**Genetic and environmental modifiers influencing disease on-set**	**Organelle dysfunctions:** Mitochondria ER stress	**Genetic and environmental modifiers affecting disease progress**	**Delivery issues to the** central nervous system (CNS)
**Significance of Inflammation**	**Transcription and RNA toxicity**	**Gender issues**	**Biomarkers** for disease staging, drug tests and novel therapeutics

The table describes some of the pertinent open questions in the field of HD research. The issues are depicted according to a time-line from the initiation, to the progression and possible treatments of the disease (columns, left to right). These can help to identify the most crucial knowledge gaps to be experimentally addressed and further considered in the field.

In this review, we have discussed current animal models utilized to study basic mechanisms and the pathophysiology of HD. Work using different neurotoxins and the application of the complex genetic models have contributed significantly to our understanding of HD's neurobiology and disease progression.

Our review represents an updated look into the complex array of available rodent models, transgenic and humanized ones, with also mentioning of the emerging non-primate pig and knock-in models. In a seminal paper, dated some time back, Pouladi and colleagues have discussed the background and the logic in choosing animal models to study HD (Pouladi et al., 2013). Previous studies on HD employing models like zebrafish, worm (*Caenorhabitis elegans*), fruit fly (*Drospohila melanogaster*) for the study of molecular mechanisms have further been thoroughly covered by others (Nittari et al., 2023; Yu et al., 2024). These models are thus not discussed further here.

As concluded in this and in the previous reviews, each animal model has limitations and may not accurately reflect all aspects or neuropathological changes observed in human patients. This raises concerns about the interpretation of molecular mechanisms and initial changes associated with HD. Of the models discussed, the genetic ones hold greater potential for providing a more accurate representation of HD's pathophysiology and molecular characteristics, as well as for investigating potential therapies.

The genetically engineered KI mouse models appear relevant to the human condition of HD as they express the human mHTT gene fragment within the mouse HD locus and produce aggregates in the most affected brain areas and in peripheral tissues, similar to those observed in humans. A limitation of the KI mice is the slow onset of motor abnormalities, which could complicate rapid drug screening. Additionally, non-human primate models, such as the pig, have recently been introduced to study neuroinflammation in HD. Research utilizing these animals can in the long-run lead to novel insights into the pathophysiology and mechanisms of HD, and thereby offer clues for potential therapies in the future.

## CRediT authorship contribution statement

**Polina Stepanova:** Writing – review & editing, Writing – original draft, Data curation, Conceptualization. **Merja H. Voutilainen:** Writing – review & editing, Funding acquisition, Data curation, Conceptualization. **Ove Eriksson:** Writing – review & editing, Visualization, Data curation, Conceptualization. **Dan Lindholm:** Writing – review & editing, Funding acquisition, Data curation, Conceptualization.

## Funding

Work in our laboratories on HD has been supported by: 10.13039/501100000942Swedish Brain Foundation, 10.13039/501100004012Jane and Aatos Erkko Foundation, 10.13039/100030905Finnish Parkinson Foundation, 10.13039/501100003125Finnish Cultural Foundation, Finska Läkaresällskapet, The 10.13039/501100008397Finnish Society of Sciences and Letters, Liv och Hälsa Foundation, 10.13039/501100004155Magnus Ehrnrooth Foundation and 10.13039/501100001658Minerva Foundation.

## Declaration of competing interest

The authors declare that they have no known competing financial interests or personal relationships that could have appeared to influence the work reported in this paper.

## Data Availability

No data was used for the research described in the article.

## References

[bib1] Andreassen O.A., Dedeoglu A., Ferrante R.J., Jenkins B.G., Ferrante K.L., Thomas M., Friedlich A., Browne S.E., Schilling G., Borchelt D.R., Hersch S.M., Ross C.A., Beal M.F. (2001). Creatine increase survival and delays motor symptoms in a transgenic animal model of Huntington's disease. Neurobiol. Dis..

[bib2] Andreassen O.A., Dedeoglu A., Stanojevic V., Hughes D.B., Browne S.E., Leech C.A., Ferrante R.J., Habener J.F., Beal M.F., Thomas M.K. (2002). Huntington's disease of the endocrine pancreas: insulin deficiency and diabetes mellitus due to impaired insulin gene expression. Neurobiol. Dis..

[bib3] Beal M.F., Kowall N.W., Ellison D.W., Mazurek M.F., Swartz K.J., Martin J.B. (1986). Replication of the neurochemical characteristics of Huntington's disease by quinolinic acid. Nature.

[bib4] Beal M.F., Ferrante R.J., Swartz K.J., Kowall N.W. (1991). Chronic quinolinic acid lesions in rats closely resemble Huntington's disease. J. Neurosci..

[bib5] Beal M.F., Brouillet E., Jenkins B.G., Ferrante R.J., Kowall N.W., Miller J.M., Storey E., Srivastava R., Rosen B.R., Hyman B.T. (1993). Neurochemical and histologic characterization of striatal excitotoxic lesions produced by the mitochondrial toxin 3-nitropropionic acid. J. Neurosci..

[bib6] Beal M.F., Brouillet E., Jenkins B., Henshaw R., Rosen B., Hyman B.T. (1993). Age-dependent striatal excitotoxic lesions produced by the endogenous mitochondrial inhibitor malonate. J. Neurochem..

[bib7] Bjorkqvist M., Fex M., Renstrom E., Wierup N., Petersen A., Gil J., Bacos K., Popovic N., Li J.Y., Sundler F., Brundin P., Mulder H. (2005). The R6/2 transgenic mouse model of Huntington's disease develops diabetes due to deficient beta-cell mass and exocytosis. Hum. Mol. Genet..

[bib8] Bjorkqvist M., Wild E.J., Thiele J., Silvestroni A., Andre R., Lahiri N., Raibon E., Lee R.V., Benn C.L., Soulet D., Magnusson A., Woodman B., Landles C., Pouladi M.A., Hayden M.R., Khalili-Shirazi A., Lowdell M.W., Brundin P., Bates G.P., Leavitt B.R., Moller T., Tabrizi S.J. (2008). A novel pathogenic pathway of immune activation detectable before clinical onset in Huntington's disease. J. Exp. Med..

[bib9] Bode F.J., Stephan M., Suhling H., Pabst R., Straub R.H., Raber K.A., Bonin M., Nguyen H.P., Riess O., Bauer A., Sjoberg C., Petersen A., von Horsten S. (2008). Sex differences in a transgenic rat model of Huntington's disease: decreased 17beta-estradiol levels correlate with reduced numbers of DARPP32+ neurons in males. Hum. Mol. Genet..

[bib10] Brouillet E., Hantraye P., Ferrante R.J., Dolan R., Leroy-Willig A., Kowall N.W., Beal M.F. (1995). Chronic mitochondrial energy impairment produces selective striatal degeneration and abnormal choreiform movements in primates. Proc. Natl. Acad. Sci. U. S. A..

[bib11] Cao C., Temel Y., Blokland A., Ozen H., Steinbusch H.W., Vlamings R., Nguyen H.P., von Horsten S., Schmitz C., Visser-Vandewalle V. (2006). Progressive deterioration of reaction time performance and choreiform symptoms in a new Huntington's disease transgenic ratmodel. Behav. Brain Res..

[bib12] Cepeda-Prado E., Popp S., Khan U., Stefanov D., Rodriguez J., Menalled L.B., Dow-Edwards D., Small S.A., Moreno H. (2012). R6/2 Huntington's disease mice develop early and progressive abnormal brain metabolism and seizures. J. Neurosci..

[bib13] Chen Y., Guillemin G.J. (2009). Kynurenine pathway metabolites in humans: disease and healthy States. Int. J. Tryptophan Res..

[bib14] Clemensson E.K.H., Novati A., Clemensson L.E., Riess O., Nguyen H.P. (2019). The BACHD rat model of Huntington disease shows slowed learning in a Go/No-Go-like test of visual discrimination. Behav. Brain Res..

[bib15] Clifford J.J., Drago J., Natoli A.L., Wong J.Y., Kinsella A., Waddington J.L., Vaddadi K.S. (2002). Essential fatty acids given from conception prevent topographies of motor deficit in a transgenic model of Huntington's disease. Neuroscience.

[bib16] Coyle J.T., Schwarcz R. (1976). Lesion of striatal neurones with kainic acid provides a model for Huntington's chorea. Nature.

[bib17] Crawford H.E., Hobbs N.Z., Keogh R., Langbehn D.R., Frost C., Johnson H., Landwehrmeyer B., Reilmann R., Craufurd D., Stout J.C., Durr A., Leavitt B.R., Roos R.A., Tabrizi S.J., Scahill R.I., Investigators T.-H. (2013). Corpus callosal atrophy in premanifest and early Huntington's disease. J Huntingtons Dis.

[bib18] Crook Z.R., Housman D. (2011). Huntington's disease: can mice lead the way to treatment?. Neuron.

[bib19] Davies S.W., Turmaine M., Cozens B.A., DiFiglia M., Sharp A.H., Ross C.A., Scherzinger E., Wanker E.E., Mangiarini L., Bates G.P. (1997). Formation of neuronal intranuclear inclusions underlies the neurological dysfunction in mice transgenic for the HD mutation. Cell.

[bib20] Dietrich P., Johnson I.M., Alli S., Dragatsis I. (2017). Elimination of huntingtin in the adult mouse leads to progressive behavioral deficits, bilateral thalamic calcification, and altered brain iron homeostasis. PLoS Genet..

[bib21] Dragatsis I., Levine M.S., Zeitlin S. (2000). Inactivation of Hdh in the brain and testis results in progressive neurodegeneration and sterility in mice. Nat. Genet..

[bib22] Duyao M.P., Auerbach A.B., Ryan A., Persichetti F., Barnes G.T., McNeil S.M., Ge P., Vonsattel J.P., Gusella J.F., Joyner A.L. (1995). Inactivation of the mouse Huntington's disease gene homolog Hdh. Science.

[bib23] Emerich D.F., Cain C.K., Greco C., Saydoff J.A., Hu Z.Y., Liu H., Lindner M.D. (1997). Cellular delivery of human CNTF prevents motor and cognitive dysfunction in a rodent model of Huntington's disease. Cell Transplant..

[bib24] Foster A.C., Miller L.P., Oldendorf W.H., Schwarcz R. (1984). Studies on the disposition of quinolinic acid after intracerebral or systemic administration in the rat. Exp. Neurol..

[bib25] Franciosi S., Ryu J.K., Shim Y., Hill A., Connolly C., Hayden M.R., McLarnon J.G., Leavitt B.R. (2012). Age-dependent neurovascular abnormalities and altered microglial morphology in the YAC128 mouse model of Huntington disease. Neurobiol. Dis..

[bib26] Furtado J.C., Mazurek M.F. (1996). Behavioral characterization of quinolinate-induced lesions of the medial striatum: relevance for Huntington's disease. Exp. Neurol..

[bib27] Gatto R.G., Ye A.Q., Colon-Perez L., Mareci T.H., Lysakowski A., Price S.D., Brady S.T., Karaman M., Morfini G., Magin R.L. (2019). Detection of axonal degeneration in a mouse model of Huntington's disease: comparison between diffusion tensor imaging and anomalous diffusion metrics. Magma.

[bib28] Gatto R.G., Weissmann C., Amin M., Angeles-Lopez Q.D., Garcia-Lara L., Castellanos L.C.S., Deyoung D., Segovia J., Mareci T.H., Uchitel O.D., Magin R.L. (2021). Evaluation of early microstructural changes in the R6/1 mouse model of Huntington's disease by ultra-high field diffusion MR imaging. Neurobiol. Aging.

[bib29] Gray M., Shirasaki D.I., Cepeda C., Andre V.M., Wilburn B., Lu X.H., Tao J., Yamazaki I., Li S.H., Sun Y.E., Li X.J., Levine M.S., Yang X.W. (2008). Full-length human mutant huntingtin with a stable polyglutamine repeat can elicit progressive and selective neuropathogenesis in BACHD mice. J. Neurosci..

[bib30] Griffioen K.J., Wan R., Brown T.R., Okun E., Camandola S., Mughal M.R., Phillips T.M., Mattson M.P. (2012). Aberrant heart rate and brainstem brain-derived neurotrophic factor (BDNF) signaling in a mouse model of Huntington's disease. Neurobiol. Aging.

[bib31] Gu X., Richman J., Langfelder P., Wang N., Zhang S., Banez-Coronel M., Wang H.B., Yang L., Ramanathan L., Deng L., Park C.S., Choi C.R., Cantle J.P., Gao F., Gray M., Coppola G., Bates G.P., Ranum L.P.W., Horvath S., Colwell C.S., Yang X.W. (2022). Uninterrupted CAG repeat drives striatum-selective transcriptionopathy and nuclear pathogenesis in human Huntingtin BAC mice. Neuron.

[bib32] Guidetti P., Luthi-Carter R.E., Augood S.J., Schwarcz R. (2004). Neostriatal and cortical quinolinate levels are increased in early grade Huntington's disease. Neurobiol. Dis..

[bib33] Guidetti P., Bates G.P., Graham R.K., Hayden M.R., Leavitt B.R., MacDonald M.E., Slow E.J., Wheeler V.C., Woodman B., Schwarcz R. (2006). Elevated brain 3-hydroxykynurenine and quinolinate levels in Huntington disease mice. Neurobiol. Dis..

[bib34] Guillemin G.J. (2012). Quinolinic acid, the inescapable neurotoxin. FEBS J..

[bib35] Guillemin G.J., Kerr S.J., Smythe G.A., Smith D.G., Kapoor V., Armati P.J., Croitoru J., Brew B.J. (2001). Kynurenine pathway metabolism in human astrocytes: a paradox for neuronal protection. J. Neurochem..

[bib36] Guillemin G.J., Smith D.G., Smythe G.A., Armati P.J., Brew B.J. (2003). Adv Exp Med Biol.

[bib37] Guillemin G.J., Smythe G., Takikawa O., Brew B.J. (2005). Expression of indoleamine 2,3-dioxygenase and production of quinolinic acid by human microglia, astrocytes, and neurons. Glia.

[bib38] Gutekunst C.A., Li S.H., Yi H., Mulroy J.S., Kuemmerle S., Jones R., Rye D., Ferrante R.J., Hersch S.M., Li X.J. (1999). Nuclear and neuropil aggregates in Huntington's disease: relationship to neuropathology. J. Neurosci..

[bib39] Hansson O., Petersen A., Leist M., Nicotera P., Castilho R.F., Brundin P. (1999). Transgenic mice expressing a Huntington's disease mutation are resistant to quinolinic acid-induced striatal excitotoxicity. Proc. Natl. Acad. Sci. U. S. A..

[bib40] Heindel W.C., Salmon D.P., Shults C.W., Walicke P.A., Butters N. (1989). Neuropsychological evidence for multiple implicit memory systems: a comparison of Alzheimer's, Huntington's, and Parkinson's disease patients. J. Neurosci..

[bib41] Helmlinger D., Yvert G., Picaud S., Merienne K., Sahel J., Mandel J.L., Devys D. (2002). Progressive retinal degeneration and dysfunction in R6 Huntington's disease mice. Hum. Mol. Genet..

[bib42] Hestad K., Alexander J., Rootwelt H., Aaseth J.O. (2022). The role of tryptophan dysmetabolism and quinolinic acid in depressive and neurodegenerative diseases. Biomolecules.

[bib43] Heyes M.P., Chen C.Y., Major E.O., Saito K. (1997). Different kynurenine pathway enzymes limit quinolinic acid formation by various human cell types. Biochem. J..

[bib44] Hickey M.A., Gallant K., Gross G.G., Levine M.S., Chesselet M.F. (2005). Early behavioral deficits in R6/2 mice suitable for use in preclinical drug testing. Neurobiol. Dis..

[bib45] Jellinger K.A. (2024). Mild cognitive impairment in Huntington's disease: challenges and outlooks. J. Neural Transm..

[bib46] Jia Q., Bai D., Zheng X., Zhu L., Ou K., Wang X., Tong H., Zhang Y., Wang J., Zeng J., Yan S., Li S., Li X.J., Yin P. (2023). Comparing HD knockin pigs and mice reveals the pathological role of IL-17. Cell Rep..

[bib47] Josefsen K., Nielsen M.D., Jorgensen K.H., Bock T., Norremolle A., Sorensen S.A., Naver B., Hasholt L. (2008). Impaired glucose tolerance in the R6/1 transgenic mouse model of Huntington's disease. J. Neuroendocrinol..

[bib48] Knopman D., Nissen M.J. (1991). Procedural learning is impaired in Huntington's disease: evidence from the serial reaction time task. Neuropsychologia.

[bib49] Kobal J., Melik Z., Cankar K., Strucl M. (2014). Cognitive and autonomic dysfunction in presymptomatic and early Huntington's disease. J. Neurol..

[bib50] Kohler C., Okuno E., Flood P.R., Schwarcz R. (1987). Quinolinic acid phosphoribosyltransferase: preferential glial localization in the rat brain visualized by immunocytochemistry. Proc. Natl. Acad. Sci. U. S. A..

[bib51] Koutouzis T.K., Borlongan C.V., Freeman T.B., Cahill D.W., Sanberg P.R. (1994). Intrastriatal 3-nitropropionic acid: a behavioral assessment. Neuroreport.

[bib52] Kuljis D.A., Gad L., Loh D.H., MacDowell Kaswan Z., Hitchcock O.N., Ghiani C.A., Colwell C.S. (2016). Sex differences in circadian dysfunction in the BACHD mouse model of Huntington's Disease. PLoS One.

[bib53] Lamirault C., Nguyen H.P., Doyere V., El Massioui N. (2020). Age-related alteration of emotional regulation in the BACHD rat model of Huntington disease. Gene Brain Behav..

[bib54] Li H., Li S.H., Cheng A.L., Mangiarini L., Bates G.P., Li X.J. (1999). Ultrastructural localization and progressive formation of neuropil aggregates in Huntington's disease transgenic mice. Hum. Mol. Genet..

[bib55] Lin C.H., Tallaksen-Greene S., Chien W.M., Cearley J.A., Jackson W.S., Crouse A.B., Ren S., Li X.J., Albin R.L., Detloff P.J. (2001). Neurological abnormalities in a knock-in mouse model of Huntington's disease. Hum. Mol. Genet..

[bib56] Lione L.A., Carter R.J., Hunt M.J., Bates G.P., Morton A.J., Dunnett S.B. (1999). Selective discrimination learning impairments in mice expressing the human Huntington's disease mutation. J. Neurosci..

[bib57] Lloret A., Dragileva E., Teed A., Espinola J., Fossale E., Gillis T., Lopez E., Myers R.H., MacDonald M.E., Wheeler V.C. (2006). Genetic background modifies nuclear mutant huntingtin accumulation and HD CAG repeat instability in Huntington's disease knock-in mice. Hum. Mol. Genet..

[bib58] Lugo-Huitron R., Ugalde Muniz P., Pineda B., Pedraza-Chaverri J., Rios C., Perez-de la Cruz V. (2013). Quinolinic acid: an endogenous neurotoxin with multiple targets. Oxid. Med. Cell. Longev..

[bib59] Mangiarini L., Sathasivam K., Seller M., Cozens B., Harper A., Hetherington C., Lawton M., Trottier Y., Lehrach H., Davies S.W., Bates G.P. (1996). Exon 1 of the HD gene with an expanded CAG repeat is sufficient to cause a progressive neurological phenotype in transgenic mice. Cell.

[bib114] Martin B., Golden E., Carlson O.D., Pistell P., Zhou J., Kim W., Frank B.P., Thomas S., Chadwick W.A., Greig N.H., Bates G.P., Sathasivam K., Bernier M., Maudsley S., Mattson M.P., Egan J.M. (2009). Exendin-4 improves glycemic control, ameliorates brain and pancreatic pathologies, and extends survival in a mouse model of Huntington’s disease. Diabetes.

[bib60] Mason H.D., McGavern D.B. (2022). How the immune system shapes neurodegenerative diseases. Trends Neurosci..

[bib61] McBride J.L., Ramaswamy S., Gasmi M., Bartus R.T., Herzog C.D., Brandon E.P., Zhou L., Pitzer M.R., Berry-Kravis E.M., Kordower J.H. (2006). Viral delivery of glial cell line-derived neurotrophic factor improves behavior and protects striatal neurons in a mouse model of Huntington's disease. Proc. Natl. Acad. Sci. U. S. A..

[bib62] Menalled L.B., Chesselet M.F. (2002). Mouse models of Huntington's disease. Trends Pharmacol. Sci..

[bib63] Menalled L.B., Sison J.D., Dragatsis I., Zeitlin S., Chesselet M.F. (2003). Time course of early motor and neuropathological anomalies in a knock-in mouse model of Huntington's disease with 140 CAG repeats. J. Comp. Neurol..

[bib64] Menalled L.B., Kudwa A.E., Miller S., Fitzpatrick J., Watson-Johnson J., Keating N., Ruiz M., Mushlin R., Alosio W., McConnell K., Connor D., Murphy C., Oakeshott S., Kwan M., Beltran J., Ghavami A., Brunner D., Park L.C., Ramboz S., Howland D. (2012). Comprehensive behavioral and molecular characterization of a new knock-in mouse model of Huntington's disease: zQ175. PLoS One.

[bib65] Moffitt H., McPhail G.D., Woodman B., Hobbs C., Bates G.P. (2009). Formation of polyglutamine inclusions in a wide range of non-CNS tissues in the HdhQ150 knock-in mouse model of Huntington's disease. PLoS One.

[bib66] Moroni F., Lombardi G., Moneti G., Aldinio C. (1984). The excitotoxin quinolinic acid is present in the brain of several mammals and its cortical content increases during the aging process. Neurosci. Lett..

[bib67] Morton A.J., Wood N.I., Hastings M.H., Hurelbrink C., Barker R.A., Maywood E.S. (2005). Disintegration of the sleep-wake cycle and circadian timing in Huntington's disease. J. Neurosci..

[bib68] Nishino H., Shimano Y., Kumazaki M., Sakurai T. (1995). Chronically administered 3-nitropropionic acid induces striatal lesions attributed to dysfunction of the blood-brain barrier. Neurosci. Lett..

[bib69] Nittari G., Roy P., Martinelli I., Bellitto V., Tomassoni D., Traini E., Tayebati S.K., Amenta F. (2023). Rodent models of Huntington's disease: an overview. Biomedicines.

[bib70] Novati A., Manfre G., Flunkert S., Van der Harst J.E., Homberg J.R., Wronski R., Nguyen H.P. (2020). Validation of behavioral phenotypes in the BACHD rat model. Behav. Brain Res..

[bib71] Palfi S., Ferrante R.J., Brouillet E., Beal M.F., Dolan R., Guyot M.C., Peschanski M., Hantraye P. (1996). Chronic 3-nitropropionic acid treatment in baboons replicates the cognitive and motor deficits of Huntington's disease. J. Neurosci..

[bib72] Palpagama T.H., Waldvogel H.J., Faull R.L.M., Kwakowsky A. (2019). The role of microglia and astrocytes in Huntington's Disease. Front. Mol. Neurosci..

[bib73] Perez-De La Cruz V., Elinos-Calderon D., Robledo-Arratia Y., Medina-Campos O.N., Pedraza-Chaverri J., Ali S.F., Santamaria A. (2009). Targeting oxidative/nitrergic stress ameliorates motor impairment, and attenuates synaptic mitochondrial dysfunction and lipid peroxidation in two models of Huntington's disease. Behav. Brain Res..

[bib74] Pido-Lopez J., Andre R., Benjamin A.C., Ali N., Farag S., Tabrizi S.J., Bates G.P. (2018). In vivo neutralization of the protagonist role of macrophages during the chronic inflammatory stage of Huntington's disease. Sci. Rep..

[bib75] Pierozan P., Zamoner A., Soska A.K., Silvestrin R.B., Loureiro S.O., Heimfarth L., Mello e Souza T., Wajner M., Pessoa-Pureur R. (2010). Acute intrastriatal administration of quinolinic acid provokes hyperphosphorylation of cytoskeletal intermediate filament proteins in astrocytes and neurons of rats. Exp. Neurol..

[bib76] Pouladi M., Morton A., Hayden M. (2013). Choosing an animal model for the study of Huntington's disease. Nat. Rev. Neurosci..

[bib77] Ramaswamy S., McBride J.L., Kordower J.H. (2007). Animal models of Huntington's disease. ILAR J..

[bib78] Risby-Jones G., Lee J.D., Woodruff T.M., Fung J.N. (2024). Sex differences in Huntington's disease from a neuroinflammation perspective. Front. Neurol..

[bib79] Roitberg B.Z., Emborg M.E., Sramek J.G., Palfi S., Kordower J.H. (2002). Behavioral and morphological comparison of two nonhuman primate models of Huntington's disease. Neurosurgery.

[bib80] Sanberg P.R., Calderon S.F., Giordano M., Tew J.M., Norman A.B. (1989). The quinolinic acid model of Huntington's disease: locomotor abnormalities. Exp. Neurol..

[bib81] Sawiak S.J., Wood N.I., Williams G.B., Morton A.J., Carpenter T.A. (2009). Use of magnetic resonance imaging for anatomical phenotyping of the R6/2 mouse model of Huntington's disease. Neurobiol. Dis..

[bib82] Scattoni M.L., Valanzano A., Popoli P., Pezzola A., Reggio R., Calamandrei G. (2004). Progressive behavioural changes in the spatial open-field in the quinolinic acid rat model of Huntington's disease. Behav. Brain Res..

[bib83] Schilling G., Becher M.W., Sharp A.H., Jinnah H.A., Duan K., Kotzuk J.A., Slunt H.H., Ratovitski T., Cooper J.K., Jenkins N.A., Copeland N.G., Price D.L., Ross C.A., Borchelt D.R. (1999). Intranuclear inclusions and neuritic aggregates in transgenic mice expressing a mutant N-terminal fragment of huntingtin. Hum. Mol. Genet..

[bib84] Schwarcz R., Kohler C. (1983). Differential vulnerability of central neurons of the rat to quinolinic acid. Neurosci. Lett..

[bib85] Shear D.A., Dong J., Gundy C.D., Haik-Creguer K.L., Dunbar G.L. (1998). Comparison of intrastriatal injections of quinolinic acid and 3-nitropropionic acid for use in animal models of Huntington's disease. Prog. Neuropsychopharmacol. Biol. Psychiatry.

[bib86] Shear D.A., Dong J., Haik-Creguer K.L., Bazzett T.J., Albin R.L., Dunbar G.L. (1998). Chronic administration of quinolinic acid in the rat striatum causes spatial learning deficits in a radial arm water maze task. Exp. Neurol..

[bib87] Shelbourne P.F., Killeen N., Hevner R.F., Johnston H.M., Tecott L., Lewandoski M., Ennis M., Ramirez L., Li Z., Iannicola C., Littman D.R., Myers R.M. (1999). A Huntington's disease CAG expansion at the murine Hdh locus is unstable and associated with behavioural abnormalities in mice. Hum. Mol. Genet..

[bib88] Shenoy S.A., Zheng S., Liu W., Dai Y., Liu Y., Hou Z., Mori S., Tang Y., Cheng J., Duan W., Li C. (2022). A novel and accurate full-length HTT mouse model for Huntington's disease. eLife.

[bib89] Slow E.J., van Raamsdonk J., Rogers D., Coleman S.H., Graham R.K., Deng Y., Oh R., Bissada N., Hossain S.M., Yang Y.Z., Li X.J., Simpson E.M., Gutekunst C.A., Leavitt B.R., Hayden M.R. (2003). Selective striatal neuronal loss in a YAC128 mouse model of Huntington disease. Hum. Mol. Genet..

[bib90] Sokka A.L., Putkonen N., Mudo G., Pryazhnikov E., Reijonen S., Khiroug L., Belluardo N., Lindholm D., Korhonen L. (2007). Endoplasmic reticulum stress inhibition protects against excitotoxic neuronal injury in the rat brain. J. Neurosci..

[bib91] Southwell A.L., Warby S.C., Carroll J.B., Doty C.N., Skotte N.H., Zhang W., Villanueva E.B., Kovalik V., Xie Y., Pouladi M.A., Collins J.A., Yang X.W., Franciosi S., Hayden M.R. (2013). A fully humanized transgenic mouse model of Huntington disease. Hum. Mol. Genet..

[bib92] Southwell A.L., Franciosi S., Villanueva E.B., Xie Y., Winter L.A., Veeraraghavan J., Jonason A., Felczak B., Zhang W., Kovalik V., Waltl S., Hall G., Pouladi M.A., Smith E.S., Bowers W.J., Zauderer M., Hayden M.R. (2015). Anti-semaphorin 4D immunotherapy ameliorates neuropathology and some cognitive impairment in the YAC128 mouse model of Huntington disease. Neurobiol. Dis..

[bib93] Southwell A.L., Skotte N.H., Villanueva E.B., Ostergaard M.E., Gu X., Kordasiewicz H.B., Kay C., Cheung D., Xie Y., Waltl S., Dal Cengio L., Findlay-Black H., Doty C.N., Petoukhov E., Iworima D., Slama R., Ooi J., Pouladi M.A., Yang X.W., Swayze E.E., Seth P.P., Hayden M.R. (2017). A novel humanized mouse model of Huntington disease for preclinical development of therapeutics targeting mutant huntingtin alleles. Hum. Mol. Genet..

[bib94] Stack E.C., Kubilus J.K., Smith K., Cormier K., Del Signore S.J., Guelin E., Ryu H., Hersch S.M., Ferrante R.J. (2005). Chronology of behavioral symptoms and neuropathological sequela in R6/2 Huntington's disease transgenic mice. J. Comp. Neurol..

[bib95] Stepanova P., Kumar D., Cavonius K., Korpikoski J., Sirjala J., Lindholm D., Voutilainen M.H. (2023). Beneficial behavioral effects of chronic cerebral dopamine neurotrophic factor (CDNF) infusion in the N171-82Q transgenic model of Huntington's disease. Sci. Rep..

[bib96] Turmaine M., Raza A., Mahal A., Mangiarini L., Bates G.P., Davies S.W. (2000). Nonapoptotic neurodegeneration in a transgenic mouse model of Huntington's disease. Proc. Natl. Acad. Sci. U. S. A..

[bib97] van Dellen A., Welch J., Dixon R.M., Cordery P., York D., Styles P., Blakemore C., Hannan A.J. (2000). N-Acetylaspartate and DARPP-32 levels decrease in the corpus striatum of Huntington's disease mice. Neuroreport.

[bib98] Van Raamsdonk J.M., Murphy Z., Slow E.J., Leavitt B.R., Hayden M.R. (2005). Selective degeneration and nuclear localization of mutant huntingtin in the YAC128 mouse model of Huntington disease. Hum. Mol. Genet..

[bib99] Van Raamsdonk J.M., Pearson J., Slow E.J., Hossain S.M., Leavitt B.R., Hayden M.R. (2005). Cognitive dysfunction precedes neuropathology and motor abnormalities in the YAC128 mouse model of Huntington's disease. J. Neurosci..

[bib100] Van Raamsdonk J.M., Gibson W.T., Pearson J., Murphy Z., Lu G., Leavitt B.R., Hayden M.R. (2006). Body weight is modulated by levels of full-length huntingtin. Hum. Mol. Genet..

[bib101] von Essen M.R., Hellem M.N.N., Vinther-Jensen T., Ammitzboll C., Hansen R.H., Hjermind L.E., Nielsen T.T., Nielsen J.E., Sellebjerg F. (2020). Early intrathecal T helper 17.1 cell activity in Huntington disease. Ann. Neurol..

[bib102] von Horsten S., Schmitt I., Nguyen H.P., Holzmann C., Schmidt T., Walther T., Bader M., Pabst R., Kobbe P., Krotova J., Stiller D., Kask A., Vaarmann A., Rathke-Hartlieb S., Schulz J.B., Grasshoff U., Bauer I., Vieira-Saecker A.M., Paul M., Jones L., Lindenberg K.S., Landwehrmeyer B., Bauer A., Li X.J., Riess O. (2003). Transgenic rat model of Huntington's disease. Hum. Mol. Genet..

[bib103] Wang C.E., Tydlacka S., Orr A.L., Yang S.H., Graham R.K., Hayden M.R., Li S., Chan A.W., Li X.J. (2008). Accumulation of N-terminal mutant huntingtin in mouse and monkey models implicated as a pathogenic mechanism in Huntington's disease. Hum. Mol. Genet..

[bib104] Wang G., Liu X., Gaertig M.A., Li S., Li X.J. (2016). Ablation of huntingtin in adult neurons is nondeleterious but its depletion in young mice causes acute pancreatitis. Proc. Natl. Acad. Sci. U. S. A..

[bib105] Wheeler V.C., White J.K., Gutekunst C.A., Vrbanac V., Weaver M., Li X.J., Li S.H., Yi H., Vonsattel J.P., Gusella J.F., Hersch S., Auerbach W., Joyner A.L., MacDonald M.E. (2000). Long glutamine tracts cause nuclear localization of a novel form of huntingtin in medium spiny striatal neurons in HdhQ92 and HdhQ111 knock-in mice. Hum. Mol. Genet..

[bib106] Wolfensberger M., Amsler U., Cuenod M., Foster A.C., Whetsell W.O., Schwarcz R. (1983). Identification of quinolinic acid in rat and human brain tissue. Neurosci. Lett..

[bib107] Woodman B., Butler R., Landles C., Lupton M.K., Tse J., Hockly E., Moffitt H., Sathasivam K., Bates G.P. (2007). The Hdh(Q150/Q150) knock-in mouse model of HD and the R6/2 exon 1 model develop comparable and widespread molecular phenotypes. Brain Res. Bull..

[bib108] Xu M., Moratalla R., Gold L.H., Hiroi N., Koob G.F., Graybiel A.M., Tonegawa S. (1994). Dopamine D1 receptor mutant mice are deficient in striatal expression of dynorphin and in dopamine-mediated behavioral responses. Cell.

[bib109] Yu Z.X., Li S.H., Evans J., Pillarisetti A., Li H., Li X.J. (2003). Mutant huntingtin causes context-dependent neurodegeneration in mice with Huntington's disease. J. Neurosci..

[bib110] Yu Z., Yang W., Yan S. (2024). Advantages and differences among various animal models of Huntington's disease. Ageing Neur Dis.

[bib111] Yu-Taeger L., Petrasch-Parwez E., Osmand A.P., Redensek A., Metzger S., Clemens L.E., Park L., Howland D., Calaminus C., Gu X., Pichler B., Yang X.W., Riess O., Nguyen H.P. (2012). A novel BACHD transgenic rat exhibits characteristic neuropathological features of Huntington disease. J. Neurosci..

[bib112] Zeitlin S., Liu J.P., Chapman D.L., Papaioannou V.E., Efstratiadis A. (1995). Increased apoptosis and early embryonic lethality in mice nullizygous for the Huntington's disease gene homologue. Nat. Genet..

[bib113] Zhang C., Wu Q., Liu H., Cheng L., Hou Z., Mori S., Hua J., Ross C.A., Zhang J., Nopoulos P.C., Duan W. (2020). Abnormal brain development in Huntington' disease is recapitulated in the zQ175 Knock-In mouse model. Cereb Cortex Commun.

